# Beyond the membrane: the overlooked nuclear localization of the prion protein

**DOI:** 10.1007/s12551-026-01471-6

**Published:** 2026-10-26

**Authors:** Mariana Juliani Do Amaral, Maria Heloisa Freire, Miguel Pires Medeiros Diniz Rodrigues, Yraima Cordeiro

**Affiliations:** Faculty of Pharmacy, Federal University of Rio de Janeiro, Rio de Janeiro, 21941-902 Brazil

**Keywords:** Prion protein, Prion diseases, Nuclear localization, Cellular localization, Biomolecular condensates, Nucleic acids, Protein-nucleic acid interaction

## Abstract

Although the prion protein (PrP) is best known as a glycosylphosphatidylinositol-anchored cell surface glycoprotein lacking canonical nucleic acid-binding domains, accumulating evidence indicates that it can be localized in the cytosol and nucleus and exhibits a remarkable ability to interact with both RNA and DNA molecules. A wide range of nucleic acids have been identified as PrP interactors, including cellular and viral RNAs, genomic and mitochondrial DNA, as well as highly structured nucleic acid motifs. For instance, G-quadruplexes and DNA hairpin aptamers, often display particularly high affinity for PrP. These observations suggest that PrP’s nucleic acid binding property is a potentially overlooked part of PrP biology, with possible implications for its physiological functions and pathological roles, including the formation of biomolecular condensates. While such condensates might participate in the spatiotemporal organization of PrP biological activities, their aging into less dynamic, gel- or solid-like assemblies could also contribute to pathological protein aggregation linked to prion disease pathogenesis. Herein, we review the literature about nuclear-associated PrP and discuss its molecular features relevant to nucleic-acid binding. Furthermore, we argue that PrP might harbor previously underappreciated nuclear functions as supported by microscopy and biochemical evidence reporting intranuclear and perinuclear PrP. We propose that this understudied nuclear localization of PrP might stem from the historical focus on membrane-associated PrP, and we call for renewed attention to its potential nuclear activities.

## Introduction

Prion diseases are the only infectious diseases transmitted by a protein and still not completely understandable at the molecular level. These fatal neurodegenerative diseases affect humans and mammals and show rapid progression, with about one year survival time after symptoms onset depending on disease subtype (Geschwind 2015; Zerr et al. 2024). Prionopathies are histopathologically characterized by brain scrapie PrP (PrP^Sc^) deposition, astrogliosis, and spongiform degeneration (vacuolation) (Prusiner 1998; Wadsworth and Collinge 2007). The existence and degree extent of these brain lesions vary within prion disease subtypes; albeit spongiform change is considered a classic neuropathological hallmark of most prion diseases (Geschwind 2015). Notwithstanding, spongiosis may be minimal or absent in certain forms, for example, some cases of the hereditary Fatal Familial Insomnia (Parchi et al. 1998). The causative agent of prion diseases is an aggregated protease-resistant infectious form of the cellular prion protein (PrP^C^), which is physiologically located at the neuronal plasma membrane. During the conversion of PrP^C^ into PrP^Sc^, there is an extreme conformational change from an α-helical C-terminal domain into a self-propagating non-native cross-β structure named scrapie (PrP^Sc^) (Pan et al. 1993; Kraus et al. 2021).

The neurodegenerative mechanisms of prion diseases have been extensively studied and are yet to be uncovered. Importantly, all proposed pathogenic mechanisms are ultimately dependent on PrP^C^, with disease probably arising from a combination of loss of normal PrP^C^ functions and neurotoxic gain-of-function effects associated with aggregated PrP^Sc^ (Aguzzi et al. 2008; Aguzzi and Falsig 2012; Mercer and Harris 2023; Zerr et al. 2024). Whether amorphous or fibrillar aggregates of PrP, oligomeric PrP, or PrP biomolecular condensates are the primary drivers of toxicity—or whether a combination of these assemblies contributes—remains a matter of extensive and further investigation (Do Amaral et al. 2023a, b; Mercer and Harris 2023; Caughey et al. 2025). Additionally, whether cofactors such as nucleic acids can lower the energy barrier for the conformational conversion of PrP remains an important question. Our previous studies provided initial evidence that nucleic acids can facilitate the conversion of PrP^C^ into aggregated PrP species with PrP^Sc^-like characteristics, leading us to propose that nucleic acids may facilitate this conformational transition (Cordeiro et al. 2001). Subsequent studies provided further support for this hypothesis, demonstrating that specific RNA molecules can promote the conversion of PrP^C^ into protease-resistant PrP (PrP^Res^) (Deleault et al. 2003) and, under appropriate conditions, support the generation of infectious prions from recombinant PrP (Wang et al. 2010). These findings have therefore raised the possibility that nucleic acids may function not merely as promoters of PrP aggregation, but as cofactors that facilitate conformational conversion toward an infectious state. Whether specific nucleic acids are required for prion infectivity in mammals, however, remains a matter of ongoing debate. Suggested pathological mechanisms of prionopathies include metal ions dyshomeostasis, the generation of reactive oxygen species together with altered mitochondria function and morphology, altered plasma membrane permeability, abnormal ion channels currents, aberrant autophagy/lysosomal pathways and prolonged activation of the unfolded protein response (Caughey et al. 2009; Zerr et al. 2024). The complexity of the neurodegenerative mechanisms of prion diseases might be related to its multiple binding partners (Aguzzi and Falsig 2012; Linden 2017), as well as the various subcellular locations that PrP^C^ shuttles, including the endoplasmic reticulum (ER), Golgi apparatus, endolysosomes and even the nucleus (Godsave et al. 2015; Alves et al. 2020). Although PrP^C^ is canonically found attached to the outer leaflet of the plasma membrane through a C-terminal glycosylphosphatidylinositol (GPI) anchor, endogenous PrP^C^ has also been detected in the nucleus and associated with nuclear structures under specific cellular conditions. Nuclear localization of PrP^C^ has been reported in proliferating epithelial cells (Morel et al. 2008) and in endocrine and neuronal cells, where PrP^C^ was detected in association with the nuclear lamina and interacting with nuclear structural proteins (Strom et al. 2011). Nuclear forms of PrP have also been described in the context of PrP variants associated with inherited prion diseases (Lorenz et al. 2002). In line with this, our group and others have described the in vitro binding of PrP to nucleic acids of different sequences, structures, and lengths (Cordeiro et al. 2001, 2023; Gabus et al. 2001; Nandi 2008; Cavaliere et al. 2013; Macedo and Cordeiro 2017), as well as the modulation of PrP phase transitions by DNA or RNA (Matos et al. 2020; Passos et al. 2021; Tahira et al. 2025). Moreover, nuclear localization of mutant or pathological forms of PrP has been observed in several cellular models, including stop-codon mutants such as PrP-Y145Stop and disease-associated PrP^Sc^. Together, these findings support the idea that nucleic acid interactions may represent an important yet underexplored aspect of PrP biology, potentially linking its nuclear localization, condensate formation, and pathological conversion. In this review, we summarize studies reporting nuclear-associated localization of cellular PrP, PrP constructs, and disease-associated PrP variants (Tables 1 and 2). We also discuss the putative physiological and pathological roles of nuclear PrP and highlight current knowledge regarding PrP-nucleic acid interactions.

**Table 1:** Nuclear associated localization of wild-type PrP. Compilation of studies suggesting intranuclear, perinuclear or association to the nuclear envelope of wild-type PrP constructs. Works are listed in chronological order from publication date

Wild-type PrP						
Cell line/tissue	PrP form	Detection tool	Method	Trigger	Intranuclear**?**	Reference
Human promyelocytic leukemia cells (NB4)	PrP^C^	Anti-PrP mAb^a^ (3F4)	IF^b^ confocal microscopy + Immunoblotting	ND^c^	Yes (nucleolus)	(Rybner et al. (2002)
Human neuroblastoma (M17)	PrP1-114-GFP; PrP23-114-GFP	GFP fluorescence + anti-PrP (3F4)	IF^b^ confocal microscopy	Proteasome inhibition (ALLN^d^)	Yes (nuclear accumulation)	(Gu et al. 2003)
Mouse hippocampal neurons	PrP^C^	Anti-PrP mAbs^a^ (8H4, SAF32) + Fabs	Cryo-immunogold EM^e^	ND^c^	Yes (nucleus + nuclear envelope)	(Mironov Jr. et al. 2003)
Inducible expression in mouse hippocampal or neuroblastoma Neuro2a cells (Npl1 or N2a PrP^-/-^)	PrPΔGPI	Anti-PrP mAbs^a^ (3F4, SAF32)	IF^b^ microscopy	ND^c^	Yes (in multinucleated cells)	(Crozet et al. 2006)
Baby hamster kidney cell line (BHK)	GFP-PrP; DsRed-ΔSP-PrP	GFP/DsRed fluorescence	Confocal microscopy	ND^c^	Yes (nuclear DsRed-ΔSP-PrP; perinuclear GFP-PrP)	(Nikles et al. 2008)
Proliferating human intestinal epithelial adenocarcinoma (Caco-2/TC7)	PrP^C^; GFP-PrP	GFP fluorescence + anti-PrP mAb^a^ (12F10)	IF^b^ + Immuno-EM^e^	Mitosis	Yes (granular pattern)	(Morel et al. 2008)
Tissues from CD1 mice (in vivo)	PrP^C^	Anti-PrP mAb^a^ (13F10)	IF^b^ confocal microscopy	ND^c^	No (nuclear membrane only)	(Strom et al. 2011)
Human neuroblastoma (SH-SY5Y)	PrP^C^	Anti-PrP mAb^a^ (EP1802Y)	IF^b^ confocal microscopy + PLA^g^	Genotoxic stress (MMS^f^)	Yes (DNA repair-related)	(Bravard et al. 2015)
Proliferating epithelial cells lines (Caco-2/TC7, SW480, HIEC-6)	PrP^C^	Anti-PrP mAb^a^ (12F10)	IF^b^ + PLA^g^ + IP^h^ + LC–MS/MS^i^	Mitosis	Yes	(Besnier et al. 2015)
Human lung diploid fibroblasts (WI-38)	PrP^C^	Anti-PrP mAb^a^ (4F7)	IF^b^ confocal microscopy	Long exposure to CuSO_4_	Yes	(Boilan et al. 2018)
Hippocampal neuron cells (NpL2)	Bovine PrP-EGFP	Anti-bovine PrP mAb^a^ (1D12 and T2) + GFP fluorescence	IF^b^ confocal microscopy	ND^c^	Yes (granular pattern)	(Hosokawa et al. 2008)
Pancreatic ductal adenocarcinoma (PDAC)	PrP^C^	Immunogold particles	Transmission EM^e^	ND^c^	Yes (nuclear compartment)	(Bianchini et al. 2021)
Human embryonic kidney cells (HEK293) and Henrietta Lacks adenocarcinoma cells (HeLa)	PrP-YFP-GPI	YFP fluorescence	IF^b^ confocal microscopy	Long exposure to CuCl_2_	No (perinuclear only)	(Do Amaral et al. 2023a, b)
N2a, SH-SY5Y, mouse primary cortical neurons	PrPΔGPI^j^; PrPΔGPI-GFP	Anti-PrP mAb^a^ (3F4)	IF^b^ confocal microscopy	Cellular pathways inhibitors (Apratoxin S9, Importazole, TAK-243)	Yes (nuclear + perinuclear PrP; nuclear PrPΔGPI-GFP)	(Banik et al. 2024)

^a^Monoclonal antibody

^b^Immunofluorescence

^c^Not described

^d^Calpain inhibitor i

^e^Electron microscopy

^f^Methyl methane sulfonate

^g^Proximity ligation assay

^h^Immunoprecipitation

^i^Liquid chromatography with tandem mass spectrometry

^j^PrPΔGPI: GPI-anchorless PrP construct

**Table 2:** Nuclear-associated localization of scrapie PrP or disease-related PrP mutants. Compilation of studies suggesting intranuclear, perinuclear or nuclear membrane-associated PrP^Sc^ or mutant PrP constructs. Works are listed in chronological order from publication date

Scrapie PrP or disease-related PrP mutants						
Cell line/tissue	PrP form	Detection tool	Method	Trigger	Intranuclear?	Reference
Scrapie neuroblastoma (N2a) cells	PrP^Sc^	Antiserum against PrP27-30	IF^a^ confocal microscopy	ND^b^	No (perinuclear accumulation)	(Taraboulos et al. 1990)
Scrapie N2a cells	PrP^Sc^	Antiserum against PrP27-30	IF^a^ confocal microscopy	ND^b^	Yes (nucleolus)	(Pfeifer et al. 1993)
Human CJD oligodendrocytes	PrP in CJD	Anti-PrP rabbit antisera (2 types)	IHC^c^	ND^b^	No (perinuclear deposits)	(El Hachimi et al. 1998)
M17 cells	Y145Stop	Anti-PrP mAb^d^ (3F4)	IF^a^ confocal microscopy	Proteasome inhibition (lactacystin or ALLN^e^)	Yes (nuclear accumulation)	(Zanusso et al. 1999)
N2a cells	GFP-W144Stop^f^; GFP-Q159Stop^g^	GFP fluorescence	Confocal microscopy	ND^b^	Yes (nuclear accumulation)	(Lorenz et al. 2002)
22L scrapie N2a cells	PrP^Sc^	Anti-PrP mAb^d^ (SAF61)	IF^a^ confocal microscopy	ND^b^	Yes (nuclear PrP is PK-resistant)	(Mangé et al. 2004)
22A scrapie-infected mouse brains	PrP-pSer43	Anti-phospho-PrP (S43 antiserum)	IHC^c^	Phosphorylation	Yes (nuclear PrP is PK-resistant)	(Giannopoulos et al. 2009)
22L scrapie N2a-3 cells (ScN2a-3) and Chandler infected ScN2a-3	PrP^Sc^	Anti-PrP mAbs^d^ (31C6, 44B1, 149, 132)	IF^a^ confocal microscopy	Protein-denaturing agent (guanidine thiocyanate)	No (perinuclear)	(Yamasaki et al. 2012)
Human CJD glial cells	PrP in CJD	Anti-PrP mAbs^d^ (3F4, SAF32, 8G8, 12F10)	IF^a^ confocal microscopy	ND^b^	Yes (nuclear + perinuclear)	(Fernández‐Vega et al. 2018)

^a^Immunofluorescence

^b^Not described

^c^Immunohistochemistry

^d^Monoclonal antibody

^e^Calpain inhibitor I

^f^Mouse PrP mutation equivalent to human Y145Stop and associated with the Gerstmann-Sträussler-Scheinker syndrome

^g^Mouse PrP mutation equivalent to human Q160Stop and associated with early onset dementia development

## From canonical trafficking to nuclear PrP

**Fig. 1:**
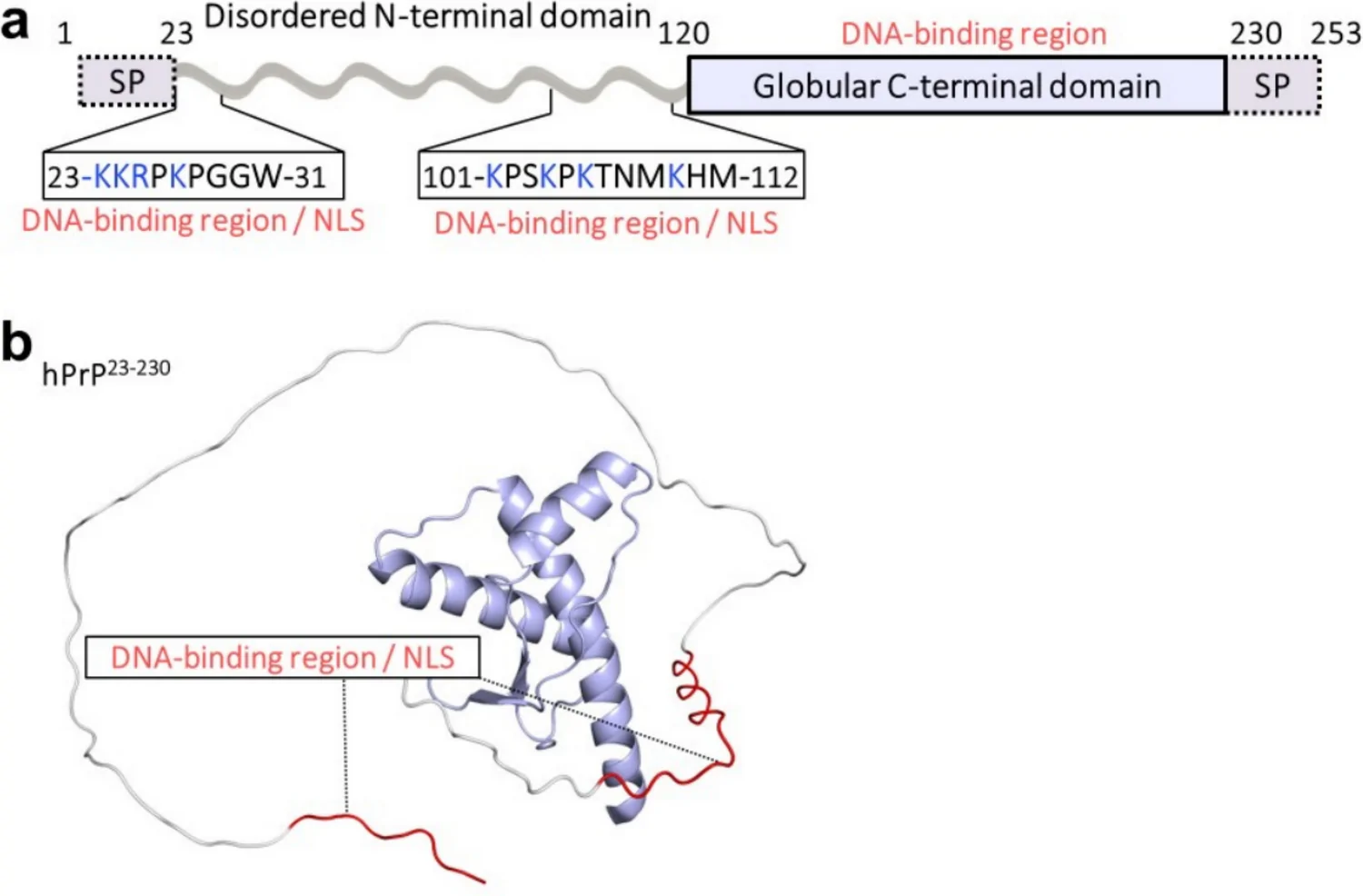
Nucleic acid-binding regions and predicted nuclear localization signals in human PrP. Domain organization of human PrP (**a**) and NMR-solved 3D structure of human PrP (PDB: 2LSB, residues 90–231) (**b**) highlighting the NLSs and nucleic acid-binding regions mapped onto the PrP structure. The N-terminal regions are shown based on an AlphaFold model

The mature cellular PrP is 208-amino acid protein (residues 23–230 of the full-length precursor) that has a long intrinsically disordered region followed by an α-helical enriched globular domain (Fig. 1a and b) (Riek et al. 1996, 1997; Donne et al. 1997). Full-length PrP is synthesized as a secretory-pathway precursor whose targeting to the endoplasmic reticulum (ER) is directed by an N-terminal signal sequence. As nascent PrP emerges from the ribosome, its N-terminal signal sequence is recognized by the signal recognition particle (SRP), promoting targeting of the ribosome-nascent chain complex to the Sec61 translocon in the ER. Subsequent translocation is regulated by the properties of the PrP signal sequence and additional translocon-associated factors, influencing the topology and efficiency of PrP ER import (Hegde et al. 1998b; Rane et al. 2008). Within the lumen of the ER, the N-terminal signal peptide is cleaved, and the protein undergoes early folding and post-translational processing, including N-linked glycosylation and disulfide bond formation (Haraguchi et al. 1989). At the C-terminus, recognition and cleavage of the GPI-addition signal enable attachment of the glycosylphosphatidylinositol anchor, thereby tethering PrP to inner luminal face of the ER membrane (Stahl et al. 1990). After ER processing, PrP traffics through the Golgi apparatus, where its glycans are further remodeled, yielding mature unglycosylated, monoglycosylated, and diglycosylated species (Rudd et al. 1999). The mature protein is then delivered to the plasma membrane, where it remains GPI-anchored at the extracellular leaflet and preferentially associates with cholesterol- and sphingolipid-enriched lipid raft domains (Campana et al. 2005; Puig et al. 2014). From the cell surface, PrP can be internalized through clathrin-mediated endocytosis or via clathrin-independent pathways, including caveolin/caveolae-associated routes, while its association with lipid rafts influences its membrane localization and subsequent trafficking through endocytic compartments (Shyng et al. 1994; Peters et al. 2003; Sunyach et al. 2003). Following internalization, PrP remains oriented toward the lumen of endocytic compartments, where its trafficking can influence signaling and determine whether the protein is recycled to the plasma membrane or targeted for lysosomal degradation. An alternative route has also been suggested, in which PrP is incorporated into multivesicular bodies and subsequently released extracellularly in exosomes or delivered to lysosomes for degradation (Campana et al. 2005; Alves et al. 2020).

Beyond the canonical secretory pathway, PrP can give rise to topologically altered protein species during/after ER trafficking due to its central hydrophobic domain (residues 112–135) that interacts with membranes. Among these, ^Ctm^PrP and ^Ntm^PrP result from alternative transmembrane orientations of the nascent polypeptide chain (Hegde et al. 1998a; Kim and Hegde 2002). These isoforms differ in the exposure of their domains to the cytosol: in ^Ctm^PrP, the N-terminal region of PrP faces the cytosol, whereas in ^Ntm^PrP this region remains within the ER lumen (Kim and Hegde 2002; Alves et al. 2020). This difference is relevant because the N-terminal domain of PrP is flexible and contains highly basic lysine-rich clusters associated with polyanionic molecules binding, including nucleic acids (Gomes et al. 2008). However, because these isoforms remain associated to the ER membrane, they are excluded from the cytosolic environment. The ^Ctm^PrP has been more directly linked to pathological outcomes, as elevated ^Ctm^PrP levels have been associated with ER stress, cell death, and neurodegenerative phenotypes, whereas the physiological or pathological relevance of ^Ntm^PrP remains less clearly defined (Hegde et al. 1998a; Kim and Hegde 2002).

CytoPrP refers to PrP forms localized in the cytosol, outside the lumen of membrane-bound components of the secretory pathway. The generation of cytoPrP can result from inefficient translocation of nascent PrP into the ER, which has been associated with the limited efficiency of the N-terminal signal peptide (Rane et al. 2004). Importantly, ER import is also influenced by structural features beyond the signal peptide. α-helical domains were shown to promote the translocation of intrinsically disordered regions into the ER (Miesbauer et al. 2009), and the C-terminal domain of PrP was demonstrated necessary and sufficient for efficient ER import (Heske et al. 2004). These findings indicate that PrP translocation depends on multiple sequence and structural determinants. CytoPrP may also arise from the retrotranslocation of misfolded ER species to the cytosol through the ER quality-control system (Ma and Lindquist 2001). In either case, cytoPrP is generally targeted to the ubiquitin–proteasome system for degradation (Ma and Lindquist 2001; Rane et al. 2004). However, during ER stress, proteasomal impairment, or pathological contexts involving disease-associated PrP mutants and/or an increased burden of misfolded PrP species, cytosolic PrP may escape efficient proteasomal degradation and accumulate (Ma and Lindquist 2001; Rane et al. 2004; Orsi et al. 2006; Kristiansen et al. 2007). This mechanism is particularly relevant to pathogenic PrP variants carrying premature stop codons within the C-terminal domain. For example, the GSS-associated Y145Stop variant is rapidly degraded by the proteasome, and inhibition of proteasomal degradation results in its intracellular accumulation, including in the nucleus (Zanusso et al. 1999).

Once in the cytosol, cytoPrP might interact with RNAs and other polyanionic ligands, mainly through its N-terminal region, which has been shown to bind nucleic acids and drive biomolecular condensation (Gomes et al. 2008; Lathe and Darlix 2020; Agarwal et al. 2021). Notably, the presence of PrP in the cytosol provides a plausible route for its nuclear import, either by active transport via NLS (importin-mediated) or passive diffusion. The existence of a possible nuclear localization signal in PrP was first appointed by Oesch (Oesch 1994) based on the enrichment of lysine and prolines of an N-terminal short motif comprising residues 23–30. Further, Jaegly and coworkers investigated whether a shorter version of this peptide (23–28) traffics to the nucleus by generating a fusion construct with the green fluorescent protein (EGFP) followed by transfection of HeLa, SK-N-SH or NT2 cells (Jaegly et al. 1998). However, they concluded that the PrP 23–28 sequence did not drive nuclear localization in any of these cell lines, in contrast to the positive control (canonical NLS of the SV40 large T antigen fused to EGFP), which showed significant nuclear localization by microscopy (Jaegly et al. 1998). Nonetheless, this study discusses two other hypotheses for nuclear PrP^C^ or PrP^Sc^: (*i*) proteins smaller than ~ 50 kDa can generally passively diffuse through the nuclear pore complexes at the nuclear membrane; hence, monomeric PrP would be able to translocate, but not aggregated PrP; (*ii*) they also raised the possibility of PrP binding to a receptor/ligand forming a complex that is further internalized and targeted to the nucleus. In line with that, PrP binds to the 37/67 kDa laminin receptor (LRP/LR), which acts as a cell-surface platform for both PrP^C^ (Gauczynski et al. 2001) and PrP^Sc^ (Gauczynski et al. 2006). Upon binding, the PrP-LRP/LR complex is internalized from the cell surface, and one study has demonstrated that LRP/LR can co-localize with PrP in the perinuclear space (Nikles et al. 2008), perhaps suggesting an escorting role towards the nucleus, although clear nuclear localization of this complex was not observed in the conditions tested in BHK cells. More recently, a parallel nuclear-targeting mechanism has been elucidated for PrP that remains in the cytosol and does not follow the secretory pathway: this cytosolic PrP is first stabilized by the chaperone VCP/p97 and then transported into the nucleus via importin-β, a process dependent on PrP’s flexible N-terminal domain (Banik et al. 2024). Once inside the nucleus, PrP might influence gene expression, and its ability to bind nucleic acids together with the high concentration of nuclear DNA/RNA might be a buffering mechanism against aberrant phase transitions.

It should be emphasized that, in the early 1990s, two independent groups had already reported nuclear-associated PrP in infected N2a cells by immunofluorescence analyzed by confocal microscopy (Taraboulos et al. 1990; Pfeifer et al. 1993). Subsequent studies explored the understanding of PrP nuclear trafficking in light of putative NLSs. In contrast to Dormont’s work (Jaegly et al. 1998), Neena Singh’s group described that extending the putative NLS peptide from 23–30 to 23–32 was crucial, as this modification enabled efficient nuclear transport in M17 neuroblastoma cells (Gu et al. 2003). Crucially, these two studies used a very similar approach by attaching putative NLSs to fluorescently labelled proteins. However, when fusing the 23–32 PrP peptide (NLS-I) to BSA-FITC, they did report nucleocytoplasmic shutting. Additionally, this study also identified another NLS, the sequence comprising residues 101–106 (KPSKPK; NLS-II) which was in fact stronger than NLS-I (95% of cells with nuclear accumulation vs. 75% for NLS-I) (Gu et al. 2003). Using an in vitro nucleocytoplasmic transport assay, they showed that both NLSs act independently and are sufficient to mediate cytosol- and ATP-dependent nuclear import in permeabilized cells. Moreover, PrP^23−114^-GFP showed strict nuclear localization in transfected cells. Alanine-scanning mutagenesis of lysine and proline residues further confirmed that NLS-I and NLS-II operate independently yet complement each other, as only their combined disruption eliminated abolished localization (Gu et al. 2003). When PrP is not retained within the secretory pathway or anchored to the membrane by a GPI moiety, these cryptic NLSs could become accessible to the nuclear import machinery and may mediate PrP translocation into the nucleus, by analogy with canonical NLS-dependent active transport (Lu et al. 2021).

On the other hand, Crozet and coworkers have demonstrated that PrP containing mutations on both NLS-I and NLS-II sites is still able to get into the nuclei from N2a cells. This observation supports the idea that PrP can passively diffuse through the nuclear membrane and bind to DNA sticking to the nuclear compartment (Crozet et al. 2006).

## Experimental evidence for physiological PrP nuclear localization

Studies summarized in Table 1 demonstrate that endogenous PrP or overexpressed PrP constructs in cell lines localize either within the nucleus, bound to the nuclear envelope, or in the perinuclear region. A wide variety of cell lines from mouse and human, under normal growth conditions, or upon diverse stressor conditions (genotoxic stress, copper treatment, proteasome inhibition) have been used as model systems in which nuclear-associated PrP is reported mainly by immunofluorescence with anti-PrP monoclonal antibodies followed by confocal microscopy.

Nuclear localization is reported in transformed cancerous cell lines, especially in actively dividing/proliferating cells and under genotoxic stress by an alkylating agent that triggers PrP nuclear localization (Table 1). Upon overnight incubation with copper ions, it has also been reported that PrP^C^ is localized in the nuclei of human fetal lung fibroblast cells (Boilan et al. 2018). Copper ions can catalyze Fenton-like reactions generating reactive oxygen species, which in turn attack DNA nucleosides (Valko et al. 2005). Similarly, genotoxic stress leads to chemical modification of macromolecules. Interestingly, it has been shown that p53, a major protein that activates different DNA repair mechanisms, directly binds to the *PRNP* promoter and upregulates its expression (Vincent et al. 2009). Therefore, these studies support a role for PrP on DNA repair.

Remarkably, removal of the GPI-anchor by expressing truncated PrP constructs promotes nuclear entry. GPI-anchorless PrP consistently shows nuclear accumulation (Crozet et al. 2006; Banik et al. 2024), whereas full-length PrP often shows perinuclear or nuclear envelope localization (Mironov Jr. et al. 2003; Nikles et al. 2008; Strom et al. 2011). It is likely that the GPI-anchor prevents PrP from nuclear trafficking as it is contained within vesicles and endolysosomes, facing the lumen of these organelles. We have previously shown that recombinant PrP undergoes phase separation in liquid-like droplets modulated by the concentration of nucleic acids (Matos et al. 2020; Tahira et al. 2025). Based on our in vitro data, high protein:NA ratios, analogous to the cytosolic milieu, results in enhanced phase separation, whereas low protein:NA molar ratios, as compared to the intranuclear environment, keeps PrP in a soluble phase (Matos et al. 2020). In line with our recombinant system, murine neuroblastoma cells (N2a) expressing GPI-anchorless PrP fused to GFP concentrates nuclear PrP as a cellular mechanism to counteract PrP aggregation (Banik et al. 2024). The excess of RNA in relation to PrP prevents its aggregation in the nucleus and facilitates clearance via the ubiquitin–proteasome pathway (Banik et al. 2024). Since anchorless PrP can exist as a product of proteolytic cleavage (“shedding”) (Glatzel et al. 2015), nuclear PrP might be functionally relevant, potentially contributing to transcriptional regulation. Indeed, PrP interacts with nuclear proteins like CBP70 (Rybner et al. 2002) and modulates the Wnt signaling pathway (Besnier et al. 2015). Furthermore, the Biogrid database (https://thebiogrid.org/) (accessed on June 15, 2026) shows many nuclear proteins annotated as physical PrP interactors, for instance, related to mRNA splicing like the CWC15 spliceosome associated protein homolog (CWC15) (Satoh et al. 2009), and many other key players in nuclear transport and nuclear structure. For instance, importins (IPO5, IPO7, IPO8, IPO9, IPO11, KPNB1) shuttle proteins into the nucleus, while lamins (LMNA, LMNB2) form the nuclear lamina, providing structural support and regulating gene expression; all these proteins were screened as PrP interactors (Satoh et al. 2009; Dittmer et al. 2014; Ulbrich et al. 2018; Shafiq et al. 2021; Li et al. 2022).

## Nucleic acid binding and putative physiological roles of nuclear PrP

Although PrP lacks canonical nucleic acid-binding domains, both wild-type soluble recombinant PrP (rPrP) and PrP^Sc^ have been shown to interact with DNA and RNA with high affinity in vitro (extensively reviewed in Silva and Cordeiro 2016; Macedo and Cordeiro 2017). A major caveat in this field is the lack of evidence for PrP-nucleic acid interaction in live cells. 

The interaction of PrP with NAs is promiscuous as it binds eukaryotic and prokaryotic NAs regardless of size and sequence, albeit with different binding kinetics and thermodynamic parameters (Gomes et al. 2008; Macedo et al. 2012; Cavaliere et al. 2013; Macedo and Cordeiro 2017; Kovachev et al. 2019; Passos et al. 2021). Recombinant PrP can also interact with simple repetitive homoribopolynucleotides (e.g., poly(A), poly(T), poly(G)) and these NAs have been reported to promote the templated-assisted conversion of PrP^C^ to PrP^Sc^ (Deleault et al. 2007; Saá et al. 2012). Interestingly, among the very few studies that have successfully generated recombinant PrP with both proteinase K resistance and infectivity in cell culture and mice, one employed a serial protein misfolding cyclic amplification (PMCA) protocol that involved mixing recombinant PrP with lipids and total liver RNA (Wang et al. 2010). This finding underscores the importance of RNA as a critical cofactor, likely essential for both structural conversion and infectivity. Accordingly, electron densities attributed to RNA have been observed in cryo-EM maps of scrapie-associated fibrils (SAFs) isolated from hamster brains infected with the 263 K scrapie strain (Bridges 2024). In addition, nucleic acids have been shown to co-purify with SAF preparations obtained from prion-infected brains (Simoneau et al. 2015).

Despite the absence of canonical NA-binding domains, there are at least four regions of PrP experimentally described to be involved in NA-binding: *(i)* residues 23–31 (Lima et al. 2006; Mercey et al. 2006; Yin et al. 2008); *(ii)* residues 51–90, corresponding to the octarepeat region (Gomes et al. 2008; Yin et al. 2008; Sheng et al. 2023); *(iii)* residues 101–110 (Lima et al. 2006; Cavaliere et al. 2013); and *(iv)* the C-terminal domain (120–231) (Rhie et al. 2003; Lima et al. 2006; Bera and Nandi 2014; Matos et al. 2020). Additionally, two monopartite NLS are suggested in the N-terminal domain, and these overlap with the two N-terminal DNA-binding regions formerly described (Jaegly et al. 1998; Gu et al. 2003) (Fig. 1a and b). Interestingly, the NLS prediction algorithm NLSExplorer predicted two NLS motifs, 23-KKRPKPG-29 and 155-HRYPNQVYYRPM-166 (Li et al. 2025). Notably, the first N-terminal motif predicted by this model overlaps with the experimentally tested NLS-I, whereas the functional relevance of the C-terminal motif remains untested.

**Fig. 2:**
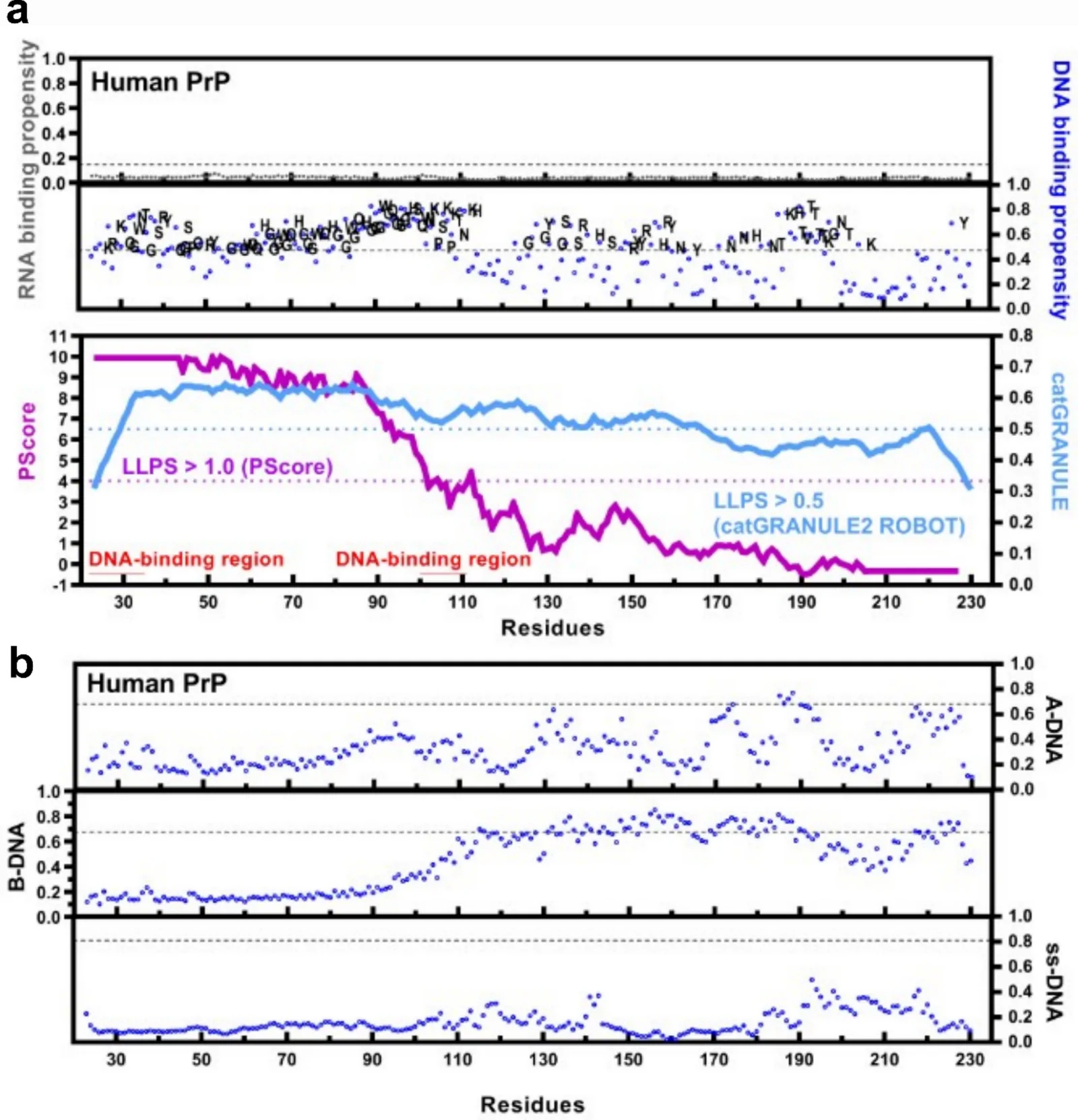
Bioinformatic prediction of nucleic acid-binding and phase-separation propensity of human PrP. (**a**, top) DRNApred analysis (Yan and Kurgan 2017) predicting RNA- (gray dots, left axis) and DNA-binding (blue dots, right axis) amino acid residues in human PrP. Letters indicate amino acids predicted to interact with nucleic acids, and dotted lines represent the corresponding prediction cutoffs. (**a**, bottom) Phase-separation propensity of PrP predicted using the PScore (Vernon et al. 2018) and catGRANULE2 ROBOT (Monti et al. 2025) algorithms, highlighting that the nucleic acid-binding regions and NLSs are embedded within the N-terminal region that drives condensation. (**b**) DNAgenie analysis (Zhang et al. 2021) predicting residues likely to interact with A-DNA, B-DNA, and single-stranded DNA

Further bioinformatics analysis using DRNApred, an algorithm that predicts NA-binding amino acid residues and discriminates between DNA- and RNA-binding residues (Yan and Kurgan 2017), showed that human PrP has a greater predicted propensity to bind DNA than RNA, with most predicted binding residues concentrated in the N-terminal region, although DNA-binding residues are also predicted in the C-terminal domain (Fig. 2a). The propensity of PrP to undergo phase separation was further assessed using the PScore (Vernon et al. 2018) and catGRANULE2 ROBOT (Monti et al. 2025) algorithms, highlighting the overlap between predicted nucleic acid-binding regions, NLSs, and the N-terminal region with a high propensity for condensation (Fig. 2a). Motivated by these predictions, we further used DNAgenie (Zhang et al. 2021), which predicts structure-annotated residues likely to interact with A-DNA, B-DNA, or single-stranded DNA. PrP was predicted to interact preferentially with B-DNA, the canonical double-stranded DNA structure (Fig. 2b).

Indeed, experimental biophysical data reveal that PrP displays a marked preference for structured nucleic acids, for instance, duplexes, hairpins, and G-quadruplexes, over their unstructured counterparts (reviewed in (Macedo and Cordeiro 2017)). These findings agree with the “structure (of RNA)-driven protein interactivity,” observed in a large study involving CLIP (Cross-Linking ImmunoPrecipitation) of protein-RNA networks (Sanchez de Groot et al. 2019). It is estimated that about 80% of ribonucleoproteins (RBPs) show bias towards binding structure-enriched RNAs. Groot et al. (2019) contextualized their results on the high dynamics of single-stranded RNAs that enable fast contacts and poor residence of proteins (Sanchez de Groot et al. 2019). On the other hand, RNA folding contributes to the existence of precise binding sites, aiding in the formation of stable protein-RNA complexes (Hackermüller et al. 2005; Sanchez de Groot et al. 2019). In line with that, we have recently identified structured RNAs from a murine cell line RNA pool that specifically bind to recombinant PrP and induce its phase transitions (Tahira et al. 2025).

These findings align with prior work from our group and others demonstrating nucleic-acid-induced PrP phase separation in vitro (reviewed in (Cordeiro et al. 2023)). Both recombinant mouse full-length PrP (rPrP^23−231^) and rPrP^90−231^, which harbors the last 30 amino acids of the N-terminus and the full C-terminal domain, undergo phase separation modulated by DNA and RNA (Matos et al. 2020; Passos et al. 2021; Tahira et al. 2025). Spectroscopic data indicate that nucleic acid binding promotes secondary and tertiary structural changes of soluble recombinant PrP, while PrP itself is suggested to promote nucleic acid unfolding, highlighting reciprocal conformational changes within PrP–NA complexes. Therefore, we hypothesize that NA conformational changes could likely be promoted inside the crowded *milieu* of PrP condensates. In summary, different NAs modulate rPrP stability in vitro; for instance, high-affinity oligonucleotides stabilize PrP^C^, whereas unspecific NAs could accelerate the conversion of PrP^C^ to PrP^Sc^ (reviewed by (Macedo and Cordeiro 2017)). Anyhow, increasing evidence suggest nucleic acids as important interactors of PrP.

## Nuclear PrP and its possible relationship to prion diseases

PrP constructs comprising truncated domains due to stop codon mutations, such as Y145stop, W144stop and Q159stop can be found inside the nucleus upon overexpression in neuroblastoma cell lines (N2a and M17) (Table 2). Among these mutants, Y145Stop is the most well-studied in relation to biophysical and biochemical properties, being directly linked to an atypical GSS phenotype (Kitamoto et al. 1993; Lorenz et al. 2002). Recently, it has been shown that the purified recombinant Y145Stop construct undergoes homotypic phase separation in a high protein and salt concentration (100 µM protein and 350 mM NaCl). However, low concentrations of transfer RNAs (50 ng/µL) significantly lower the critical concentration for phase separation in a stoichiometry-dependent mechanism, to the point in which 5 µM protein can form condensates under conditions more similar to those present in the cellular environment. These condensates formed in the presence of tRNA show rapid aging forming thioflavin T-positive fibrous-like structures, together with other amyloid-like characteristics like deep blue autofluorescence and no FRAP recovery (Agarwal et al. 2021). Several studies have also shown that RNAs are key modulators of phase transitions in prion-like proteins, including PrP mutants, acting as either dissolvers (buffering LLPS) or triggers depending on the RNA/protein molar ratio as demonstrated by a reentrant mechanism (Banerjee et al. 2017; Maharana et al. 2018; Agarwal et al. 2021; Banik et al. 2024).

Investigations into the degradation pathways of PrP show Y145Stop as a structurally unstable mutant prone to accumulate inside membrane-bound compartments. Furthermore, proteasomal inhibition with lactacystin or ALLN led to specific endoplasmic reticulum, Golgi and nuclear accumulation of PrP (Zanusso et al. 1999; Gu et al. 2003). Although the role of the suggested NLS regions in intranuclear localization of PrP is not a consensus (Crozet et al. 2006), it has been suggested that mutants with C-terminal truncation likely expose these cryptic NLS motifs, allowing the recruitment of importin-β and subsequent targeting of PrP towards the nuclei (Zanusso et al. 1999; Gu et al. 2003; Banik et al. 2024).

As for the other artificial stop codon mutants, expression of mouse PrP W144Stop and Q159Stop for 48 h resulted in restricted nuclear localization for Q159Stop, while W144Stop was found distributed throughout the cytoplasm and nucleus (Lorenz et al. 2002). As opposed to Y145Stop, these mutants do not accumulate at the Golgi apparatus and since both are also truncated, it is possible that their NLS regions mediate nucleic acid-targeting. More in depth studies of the human mutants Y145Stop and Q160Stop have revealed that their aggregates share a common fibrillar structure with a main hydrophobic core extending from residues 109–142. Within this region, residues 138–141 appear to play a crucial role in the amyloid conversion of Y145Stop (Kundu et al. 2003; Zweckstetter 2013). In parallel to this hydrophobic core, Y145Stop still possesses a high net positive charge that allows interaction with nucleic acids that can further modulate its phase transitions (Agarwal et al. 2021).

Regarding scrapie PrP, it has been described that about 10% of scrapie-infected cells (ScN2a) with the 22L strain were able to translocate most of their glycosylated PrP^Sc^ into the nucleus. This trafficking event was microtubule-dependent yet occurs via a proteasome-independent pathway (Mangé et al. 2004). Interestingly, treatment with EDTA releases these molecules from the nuclei (Mangé et al. 2004), suggesting a new role yet to be investigated for divalent cation stabilization of PrP-DNA interaction. Notably, reports of perinuclear 22L PrP^Sc^ observed co-localization with endosomal and lysosomal markers related to the Endocytic Recycling Compartment (ERC) in the intracellular pathway of transferrin, fostering new insights into the research on PrP^Sc^-metal ion interaction (Yamasaki et al. 2012). Recently, proteinase K–resistant PrP showing nuclear and perinuclear immunoreactivity in oligodendrocytes of the frontal cortex was observed in a single case of otherwise typical sporadic Creutzfeldt–Jakob disease (sCJD) type VV2a (Fernández‐Vega et al. 2018). The nucleocytoplasmic trafficking of disease-related PrP has yet to be resolved. Critically, answering this question will depend on the development of temporally inducible models that capture the heterogeneity of distinct PrP mutants or PrP strains.

## Concluding remarks

In this review, we examined the experimental evidence supporting the possible nuclear localization of the prion protein (PrP), with particular emphasis on studies based on co-localization assays, using confocal microscopy, and immunogold electron microscopy. While the intranuclear localization of several disease-associated and engineered PrP mutants has been consistently reported, the existence of a *bona fide* nuclear pool of wild-type PrP remains a matter of debate.

A major limitation is that most studies reporting nuclear localization were performed before the widespread availability of current high-resolution imaging techniques, which are now capable of more definitively distinguishing intranuclear from perinuclear or nuclear envelope-associated localizations. Since the first report describing nuclear localization of scrapie PrP in 1993 (Pfeifer et al. 1993), numerous studies have provided evidence suggesting that either cellular PrP or aberrant forms of PrP may access the nuclear compartment under specific cellular conditions (Tables 1 and 2). However, differences in experimental systems, cell types, PrP constructs, detection methods, and image resolution have often complicated the interpretation of these findings and hindered the establishment of a clear consensus. Therefore, it remains to be fully resolved whether cellular PrP exerts physiological functions within the nucleus or in the perinuclear space. Nevertheless, this question deserves renewed attention considering the growing evidence that PrP interacts with a broad range of RNA and DNA molecules and might function as a nucleic acid-binding protein (reviewed by (Cordeiro et al. 2023)). In recent years, biomolecular condensates have emerged as key regulators of nuclear organization and gene expression, with RNA and DNA playing central structural and regulatory roles in these assemblies. Given the documented ability of PrP to interact with nucleic acids and to undergo phase transitions under specific conditions, the possibility that PrP participates in, regulates, or is recruited to nuclear condensates represents an intriguing avenue for future investigation. Such a framework could provide new mechanistic insights linking nucleic acid binding, phase behavior, and the diverse physiological and pathological functions attributed to PrP.

Biophysical studies on the lifetime, association and dissociation rates, and the stability of different PrP-NA complexes are needed to understand the effect of specific NAs on PrP misfolding and phase transitions. Furthermore, even though recombinant PrP shows similar values of dissociation equilibrium constants (k_D_) with different NAs in vitro (usually in the nano- to micromolar range), in the crowded and confined environment of cells, small changes in the affinity, influenced by ionic currents and competing molecular partners, might directly influence the assembly/disassembly of PrP-NA complexes.

Re-examining PrP subcellular localization using state-of-the-art imaging approaches, combined with complementary biochemical, biophysical, and cell biology methods, may therefore provide important insights into the physiological roles of cellular PrP. Determining whether PrP is associated with specific nuclear compartments, chromatin-associated assemblies, or RNA-rich condensates may help clarify whether its nuclear presence reflects a *bona fide* biological function or a context-dependent cellular response.

By compiling and critically analyzing the available literature, we hope this review stimulates further discussion regarding the experimental approaches employed, the PrP constructs investigated, and the interpretation of nuclear localization data. More broadly, we aim to highlight unresolved questions concerning the subcellular distribution of both cellular PrP and disease-associated PrP variants, encouraging future studies that can clarify the biological significance of these observations and their potential connections to nucleic acid biology and biomolecular condensation.

## Data Availability

The bioinformatics analysis supporting the findings of this study are available within the paper.
